# Contribution of the gp120 V3 loop to envelope glycoprotein trimer stability in primate immunodeficiency viruses

**DOI:** 10.1016/j.virol.2018.06.005

**Published:** 2018-08

**Authors:** Dane Bowder, Haley Hollingsead, Kate Durst, Duoyi Hu, Wenzhong Wei, Joshua Wiggins, Halima Medjahed, Andrés Finzi, Joseph Sodroski, Shi-Hua Xiang

**Affiliations:** a Nebraska Center for Virology, University of Nebraska-Lincoln, Lincoln, NE 68583, United States; b School of Biological Sciences, University of Nebraska-Lincoln, Lincoln, NE 68583, United States; c School of Veterinary Medicine and Biomedical Sciences, University of Nebraska-Lincoln, Lincoln, NE 68583, United States; d Centre de Recherche du CHUM, Department of Microbiology, Infectious Diseases and Immunology, Université de Montréal, Montreal, QC, Canada; e Department of Cancer Immunology and Virology, Dana-Farber Cancer Institute, United States; f Department of Microbiology and Immunobiology, Division of AIDS, Harvard Medical School, Boston, MA 02215, United States; g Department of Immunology and Infectious Diseases, Harvard T.H. Chan School of Public Health, Boston, MA 02115, United States

**Keywords:** Hydrophobic patch, V3 loop, gp120, HIV-1, HIV-2, Simian immunodeficiency virus (SIV), Primate immunodeficiency viruses (PIV)

## Abstract

The V3 loop of the human immunodeficiency virus type 1 (HIV-1) gp120 exterior envelope glycoprotein (Env) becomes exposed after CD4 binding and contacts the coreceptor to mediate viral entry. Prior to CD4 engagement, a hydrophobic patch located at the tip of the V3 loop stabilizes the non-covalent association of gp120 with the Env trimer of HIV-1 subtype B strains. Here, we show that this conserved hydrophobic patch (amino acid residues 307, 309 and 317) contributes to gp120-trimer association in HIV-1 subtype C, HIV-2 and SIV. Changes that reduced the hydrophobicity of these V3 residues resulted in increased gp120 shedding and decreased Env-mediated cell-cell fusion and virus entry in the different primate immunodeficiency viruses tested. Thus, the hydrophobic patch is an evolutionarily conserved element in the tip of the gp120 V3 loop that plays an essential role in maintaining the stability of the pre-triggered Env trimer in diverse primate immunodeficiency viruses.

## Highlights

• The V3-loop of HIV-1 gp120 contributes to Env trimer stability and viral entry.
• The hydrophobic patch in the tip of the V3 loop is critical for pre-triggered Env trimer stability.
• The hydrophobic patch is a conserved motif in primate immunodeficiency viruses.

## 1 Introduction

The third variable (V3) loop of the gp120 envelope glycoprotein (Env) on the surface of human immunodeficiency virus type 1 (HIV-1) becomes exposed after the virus binds CD4, the initial receptor; the V3 loop plays an important role in contacting the coreceptor CCR5 or CXCR4 to mediate viral entry (Cashin et al., 2013, Foda et al., 2001, Hartley et al., 2005, Hongjaisee et al., 2017, Huang et al., 2005, Jiang et al., 2010, Moore and Nara, 1991). The sequence of the V3 loop determines HIV-1 tropism, dictating the choice of the coreceptor, CCR5 or CXCR4 (Cardozo et al., 2007, Cashin et al., 2013, Conley et al., 1994, Kumar and Raghava, 2013, Scheib et al., 2006, Svicher et al., 2011). The V3 loop is also immunogenic, and neutralizing antibodies against V3 often dominate immune responses following Env immunization (Jacob et al., 2015, Julien et al., 2013b, Li et al., 2015, Sirois et al., 2007, Stanfield and Wilson, 2005, Totrov et al., 2010, Watkins et al., 2011, Zolla-Pazner, 2005). In addition to its role in HIV-1 entry and immunogenicity, the V3 loop contributes to the non-covalent association of gp120 with the Env trimer, during the assembly of the viral Env spike (Xiang et al., 2010). In primary HIV-1 strains, the length of the V3 loop is extremely constrained by this assembly requirement; even the insertion of a single glycine residue in the V3 stem leads to destabilization of the Env trimer and shedding of gp120 (Xiang et al., 2010). A hydrophobic patch located in the V3 tip region has been shown to be critical for envelope trimer stability in HIV-1 subtype B strains (Xiang et al., 2010). A single-residue change to a hydrophilic or even to a neutral residue in this hydrophobic patch destabilizes the Env trimer and results in an increase of gp120 shedding, a decrease of Env-mediated membrane fusion and a reduction in virus infectivity. In available structural models of the HIV-1 Env trimer (Julien et al., 2013a, Lee et al., 2016, Lyumkis et al., 2013, Pancera et al., 2014), the V3 loop is located at the apex of the trimer along with the gp120 V1 and V2 variable regions. This apical location potentially allows the V3 loop to participate in interprotomer contacts that contribute to Env trimer stability. For example, substitution of hydrophobic residues near the V3 hydrophobic patch raised the melting temperature of soluble gp140 SOSIP.664 Env trimers (de Taeye et al., 2018, de Taeye et al., 2015).

It is not known whether the V3 contribution to Env trimer stability is specific for HIV-1 subtype B strains or also applies to other HIV-1 subtypes or evolutionarily related primate lentiviruses (Hirsch et al., 1995, Sharp and Hahn, 2011). Based on sequence alignment of the V3 region, the hydrophobic patch appears to be well conserved in HIV-1, HIV-2 and the simian immunodeficiency viruses (SIVs), implying an important function. However, given the differences between HIV-1 and HIV-2/SIV with respect to the length and composition of the V1/V2 region (Bohl et al., 2013), the structural organization of the Env trimer apex of these virus lineages may differ. Therefore, in this study, we examined the role of the V3 loop hydrophobic patch and V3 loop length in both HIV-1 and HIV-2 species, as well as in nonhuman primate lentiviruses, SIVs. Our results support the hypothesis that the V3 loop plays an important role in the stability of the unliganded HIV-1, HIV-2 and SIV Env trimers in addition to its role in contacting the coreceptor for viral entry in the CD4-bound state of Env.

## 2 Results

### 2.1 A single glycine residue insertion in the gp120 V3 loop destabilizes Env trimers from Clade C HIV-1

**Fig. 1:**
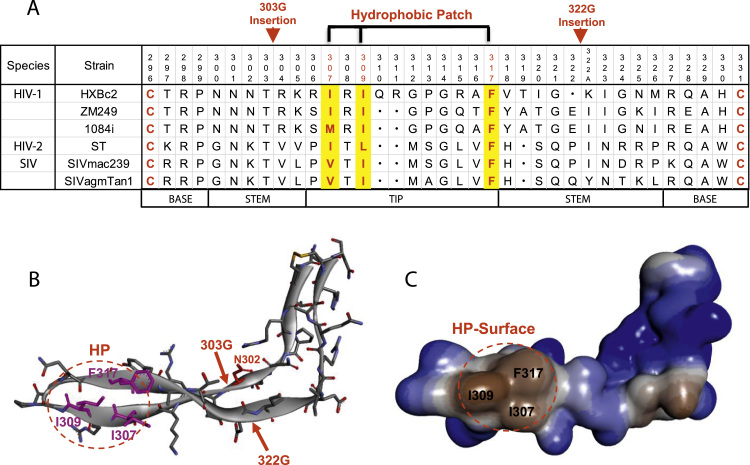
**The gp120 V3 hydrophobic patch.** (A). Alignment of the V3 loop sequences of primate immunodeficiency viruses. The amino acid residues are numbered according to standard HIV-1 HXBc2 numbering, which is included as a reference (Korber et al., 1998). The hydrophobic patch residues that were altered in the study are shown in yellow, and the locations of glycine insertions are indicated by an arrow. (B). Ribbon structure of the V3 loop in the sgp140 SOSIP.664 Env trimer (PDB: 4ZMJ). HP, hydrophobic patch indicated by the red circle. (C). The surface of the V3 loop in (B), showing the hydrophobic patch in the red circle. gray, hydrophobic, blue, hydrophilic.

**Fig. 2:**
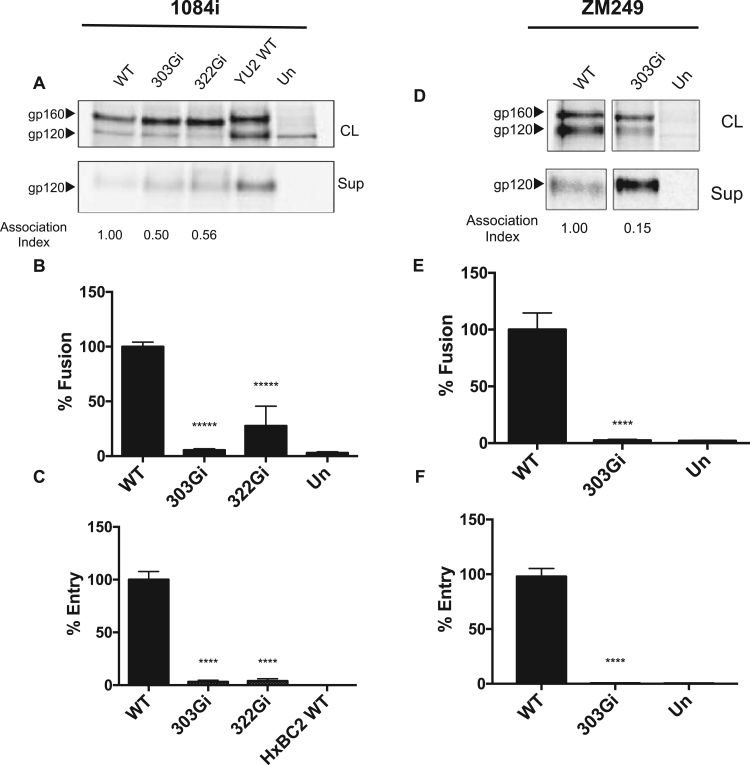
**Characterization of V3 glycine-insertion mutants of HIV-1 subtype C strains (1084i and ZM249).** (A, D). SDS-PAGE analysis of immunoprecipitated cell lysates and supernatant fractions from cells expressing WT or mutant envelope proteins. The gp160 and gp120 bands are indicated. The association index provides an indication of the non-covalent association of gp120 with the Env trimer, relative to that of the wild-type Env, and was calculated as previously reported (Finzi et al., 2010). YU2 is a subtype B HIV-1 strain used as a positive control. (B, E) Cell-cell fusion assay. (C, F) Viral entry assay. HXBc2 is an X4 tropic subtype B HIV-1 virus that was used as a negative control for viral entry. All the values of the mutants were normalized as percentages of those obtained with their respective wild-type (WT) Env, as described in the Materials and Methods. The results shown are representative of three separate experiments performed in quadruplicate. Significance was determined by the student's T-test [* ** *p < 0.001, * ** **p < 0.0001]. Un, untransfected negative-control cells.

Although the length of the gp120 V3 loop is relatively conserved at about 35 amino acids in the primate lentiviruses, there is some variation among viral species and strains (Fig. 1A). Surprisingly, in some HIV-1 strains, a single glycine residue insertion in the V3 loop can be lethal to the virus, a phenomenon that was first observed in primary HIV-1 subtype B viruses (Xiang et al., 2010). The Glycine (G) insertions were positioned between residues 303 and 304 or between residues 322 and 323; these insertions involve the variable stem region of V3 and avoid known functional regions in the loop (e.g., coreceptor-binding elements) (Fig. 1A and B). To evaluate whether this phenomenon is specific to subtype B strains or also applies to other HIV-1 subtypes or other primate lentiviruses, we investigated additional viruses, including HIV-1 subtype C, HIV-2 and SIV. HIV-1 subtype C is the dominant clade worldwide (Cashin et al., 2013, Jiang et al., 2010). To represent HIV-1 subtype C, two Zambian isolates, 1084i and ZM249M_B10 (ZM249), were studied. Radiolabeled Envs from lysates and supernatants of expressing cells were assessed, allowing an evaluation of the efficiency of Env precursor processing and gp120 association with the trimer. When a glycine residue was inserted into the V3 loop at the 303 or 322 positions, an increase in gp120 shedding was observed (Fig. 2A, D). We assessed the ability of the glycine-insertion mutants to mediate the fusion of Env-expressing cells with CD4^+^CCR5^+^ cells, and to complement the entry of an *env*-defective reporter virus into TZM-bl cells. The insertion of a glycine residue in the V3 loop significantly decreased Env-mediated cell-cell fusion and resulted in a total loss of viral infectivity (Fig. 2B, C, E, F). These data suggest that in Clade C HIV-1, as was previously observed in HIV-1 subtype B strains, the V3 loop contributes to the non-covalent association of gp120 with the pre-triggered Env trimer.

### 2.2 Role of the V3 loop hydrophobic patch in HIV-1 subtype C

**Fig. 3:**
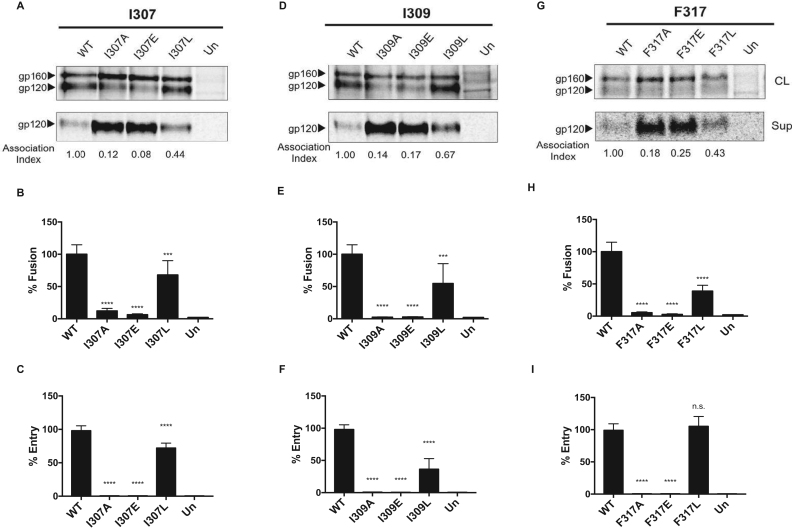
**Characterization of hydrophobic-patch mutants of HIV-1 subtype C strain ZM249.** (A, D, G). Immunoprecipitated cell lysates (CL) and supernatants (Sup) of WT HIV-1 ZM249 Env and Env mutants with alanine (A), glutamic acid (E) and leucine (L) substitutions at three hydrophobic V3 loop positions (307, 309 and 317). The association index value was calculated as previously reported (Finzi et al., 2010). (B, E,H). Cell-cell fusion assays. (C, F,I). Viral entry assays. Un, untransfected cells. Significance was determined by the student's T-test [* **p < 0.001, * ** *p < 0.0001]; n.s., not significant.

**Fig. 4:**
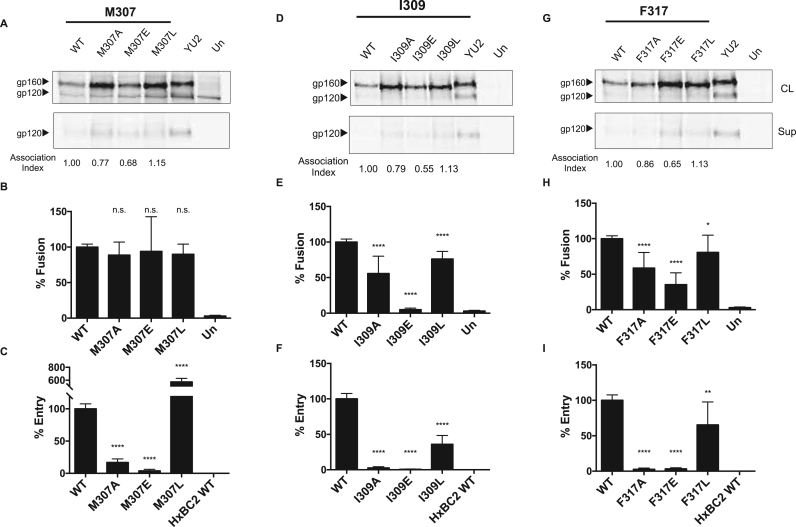
**Characterization of hydrophobic-patch mutants of HIV-1 subtype C strain 1084i.** (A, D,G). Immunoprecipitated cell lysates (CL) and supernatants (Sup) of WT HIV-1 1084i Env and Env mutants with alanine (A), glutamic acid (E) and leucine (L) substitutions at three hydrophobic V3 loop positions (307, 309 and 317). The association index value was calculated as previously reported (Finzi et al., 2010). YU2 is an Env from a subtype B HIV-1 strain used as a positive control. (B, E,H). Cell-cell fusion assays. (C, F,I). Viral entry assays. Un, untransfected cells. Significance was determined by the student's T-test [* p < 0.05, * *p < 0.01, * ** *p < 0.0001]; n.s., not significant.

Three hydrophobic residues at positions 307, 309 and 317 constitute a hydrophobic surface patch in available structures of the gp120 V3 loop (Xiang et al., 2010) (Fig. 1, B and C). Based on sequence alignment, this patch appears to be conserved in all primate lentiviruses; although some variability is tolerated, residues 307, 309 and 317 are consistently hydrophobic (Fig. 1A). We investigated the effect of specific changes in the hydrophobic patch in HIV-1 subtype C, using the two Zambian strains ZM249 and 1084i. Three different substitutions were made in each residue (307, 309 and 317) of the hydrophobic patch: a relatively neutral alanine (A) residue, a hydrophilic glutamic acid (E) residue or another hydrophobic residue, leucine (L). The results for ZM249 and 1084i are shown in Fig. 3, Fig. 4, respectively. It is evident that one hydrophilic (to E) or neutral amino acid (to A) substitution in any of the three residues of the V3 hydrophobic patch of the ZM249 Env resulted in Env trimer destabilization and increased shedding of gp120 (Fig. 3A, D, G). Substitution of the hydrophobic leucine (L) residue resulted in less dramatic increases in gp120 shedding. Decreases in gp120 association with the Env trimer also resulted from the alanine and glutamic acid substitutions in residues 307, 309 and 317 of the 1084i Env, although the magnitude of these decreases was less than that seen in the ZM249 Env. Similar to the results with the ZM249 Env, the leucine substitution resulted in a phenotype that was closer to that of the wild-type Env (Fig. 4A, D, G). These results support a model in which the hydrophobicity of the V3 patch contributes to its function in maintaining the stability of the Env trimer.

Consistent with the decrease in stability of the Env trimer, the alanine and glutamic acid substitution mutants of ZM249 Env, and to a lesser extent of the 1084i Env, exhibited a decrease in cell-cell fusion (Fig. 3B, E, H and Fig. 4B, E, H). The leucine substitution mutants were generally more capable of mediating cell-cell fusion than the alanine and glutamic acid substitution mutants.

Complementation of virus entry was severely compromised by alanine and glutamic acid substitutions in all three residues of the hydrophobic V3 patch (Fig. 3C, F, I and Fig. 4C, F, I). As has been seen for other HIV-1 Env mutants with altered subunit association (Finzi et al., 2010), virus infectivity was compromised to a greater degree than syncytium-forming ability. The leucine substitution mutants retained varying degrees of infectivity. The F317L mutant of ZM249 exhibited wild-type levels of infectivity. The M307L mutant of the 1084i Env supported virus entry significantly better than the wild-type 1084i Env; apparently the methionine residue at 307, which is less common among HIV-1 strains, is not as optimal for virus infectivity as the leucine residue. Changes in viral infection and cell-cell fusion also do not appear to be due to reduced CD4 binding, as alanine substitution mutants in ZM249 exhibited no differences in affinity to CD4-Ig (Table 1). Together, the ZM249 and 1084i Env mutant phenotypes support a role for the hydrophobic V3 patch in the stability and function of Envs from Clade C HIV-1 strains.

**Table 1:** Phenotypes of primate immunodeficiency virus mutants.

**Env and mutation**	**Association index*****a***	**Cell-cell fusion (Percent)*****b***	**Viral entry (Percent)*****c***	**CD4 Ig binding*****d***
**1084i**				
WT	1.00	100.00	100.00	n.d.
303Gi	0.50	5.40 *	3.19 *	n.d.
322G-	0.56	27.57 *	3.93 *	n.d.
M307A	0.77	88.68	16.91 *	n.d.
M307E	0.68	93.94	3.64 *	n.d.
M307L	1.15	89.81 *	573.49 *	n.d.
I309A	0.79	55.72 *	2.53 *	n.d.
I309E	0.55	4.97 *	0.66 *	n.d.
I309L	1.13	76.15 *	35.93 *	n.d.
F317A	0.86	58.57 *	2.70 *	n.d.
F317E	0.65	35.26 *	3.37 *	n.d.
F317L	1.13	80.68 *	65.28 *	n.d.
**ZM249M-B10**				
WT	1.00	100.00	100.00	1.00
303Gi	0.15	2.47 *	0.56 *	0.98
I307A	0.08	12.17 *	0.58 *	0.97
I307E	0.05	6.15 *	0.61 *	n.d.
I307L	0.29	67.91 *	72.06 *	n.d.
I309A	0.08	2.45 *	0.66 *	1.03
I309E	0.13	2.78 *	0.50 *	n.d.
I309L	0.82	54.70 *	36.31 *	n.d.
F317A	0.13	5.27 *	0.56 *	0.90
F317E	0.21	2.68 *	0.52 *	n.d.
F317L	0.40	38.76 *	105.17	n.d.
**SIVmac239**				
WT	1.00	100.00	100.00	1.00
303Gi	0.14	11.55 *	0.96 *	0.71
V307A	0.19	49.86 *	1.07 *	1.03
I309A	0.48	99.36	5.01 *	0.92
F317A	0.14	33.45 *	1.18 *	0.89
**SIVagmTan1**				
WT	1.00	100.0	n.d	n.d.
303Gi	0.45	30.20 *	n.d	n.d.
V307A	0.41	41.31 *	n.d	n.d.
I309A	0.46	46.92 *	n.d	n.d.
F317A	0.53	51.46 *	n.d	n.d.
**HIV-2 ST**				
WT	1.00	100.0	n.d	1.00
303Gi	0.43	5.96 *	n.d	0.65
I307A	0.33	8.29 *	n.d	1.03
L309A	0.34	5.06 *	n.d	0.67
F317A	0.37	6.30 *	n.d	0.63

Table 1. *a.* The association index is a measure of the envelope stability as indicated by the amount of radiolabeled WT or mutant gp120 shed into the media. The association index was calculated as follows: ([mutant gp120]_cell_ x [wild-type gp120]_supernatant_)/([mutant gp120]_supernatant_ x [wild-type gp120]_cell)_. The association index of the mutants is shown as a proportion of the WT env, which is set to 1.0.

*b.* Cell-cell fusion was assessed by coincubation of 293 T cells expressing WT or mutant env and Tat with TZMbl target cells in a ratio of 1:1, and luciferase was measured. Cell-cell fusion values of the respective WT strains were set to 100%, and mutants were expressed as a percentage of the WT value. * , indicates significant difference compared to WT Env, as determined by student's T-test (p < 0.05).

*c.* Viral entry was measured as by infecting CD4 +CCR5 + cells with replication deficient viruses expressing WT or mutant envs. Luciferase was measured as an indication of viral entry. Viral entry values of WT viruses were set to 100% and the values for the mutants are shown as a percentage of the WT value. * , indicates significant difference compared to WT, as determined by student's T-test (p < 0.05).

*d.* CD4-Ig binding of WT gp120 was established by comparing the quantity of cell supernatant bound by CD4-Ig to the total gp120 immunoprecipitated by infected host serum; values for the mutants were compared to this value. n.d. not determined.

### 2.3 Role of the V3 loop hydrophobic patch in HIV-2

**Fig. 5:**
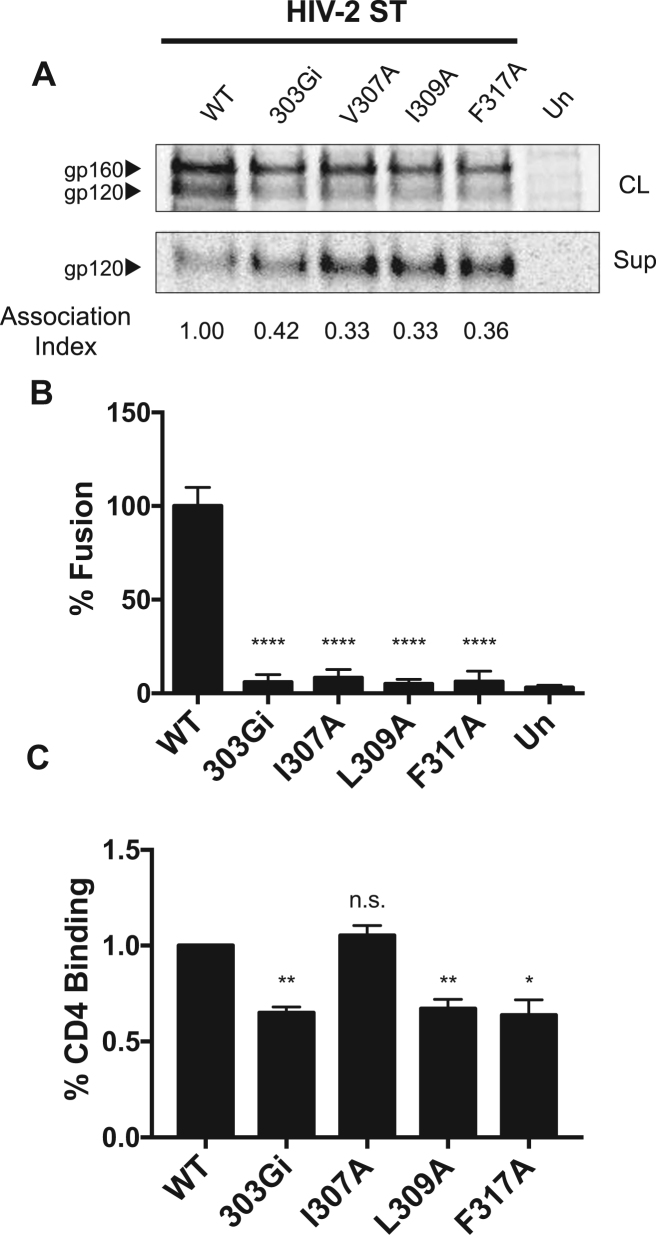
**Characterization of V3 hydrophobic-patch and glycine-insertion mutants of the HIV-2 ST strain.** (A). Immunoprecipitated cell lysates (CL) and supernatants (Sup) of WT HIV-2 ST Env and glycine-insertion (303Gi) and hydrophobic patch mutants. The association index value was calculated as previously reported (Finzi et al., 2010). (B). Cell-cell fusion assay. Un, untransfected effector cells. (C). CD4-binding assay. CD4-Ig was used to precipitate the indicated gp120 glycoproteins in transfected cell supernatants. Un, untransfected cells. The binding ratio of the mutants was compared to the binding ratio of the WT Env, as described in Materials and Methods. The results in this figure are representative of those obtained in three separate binding experiments. Significance was determined by the student's T-test [*p < 0.05, * *p < 0.01, * ** *p < 0.0001]; n.s., not significant.

Human immunodeficiency virus type 2 (HIV-2) evolved from SIVsmm species and thus represents a separate lineage from the HIV-1 M group, which is derived from SIVcpz viruses. Despite this, the hydrophobic patch is also well preserved in the gp120 V3 loops of HIV-2 species, based upon Env sequence alignment (Fig. 1A). To assess the role of the hydrophobic patch in HIV-2, we chose a typical HIV-2 strain, HIV-2 ST, to test. Alanine residues were substituted for the hydrophobic patch residues at positions 307, 309 and 317. All three substitutions caused a significant increase in gp120 shedding (Fig. 5A). This decrease in Env stability also correlated with a decrease in cell-cell fusion capability. All three alanine substitutions significantly reduced Env-mediated cell-cell fusion to background levels (Fig. 5B). Additionally, a glycine insertion in the HIV-2 ST V3 loop (303Gi) also resulted in an increase of gp120 shedding and a reduction in cell-cell fusion. The I307A change did not affect the ability of Env to bind CD4-Ig, but the alanine substitutions at positions 309 and 317 were associated with a modest decrease in CD4-Ig binding (Fig. 5C). Overall, these data suggest that the structural role and biological function of the V3 hydrophobic patch is conserved in HIV-2 species.

### 2.4 Role of the V3 loop hydrophobic patch in nonhuman primate lentiviruses

**Fig. 6:**
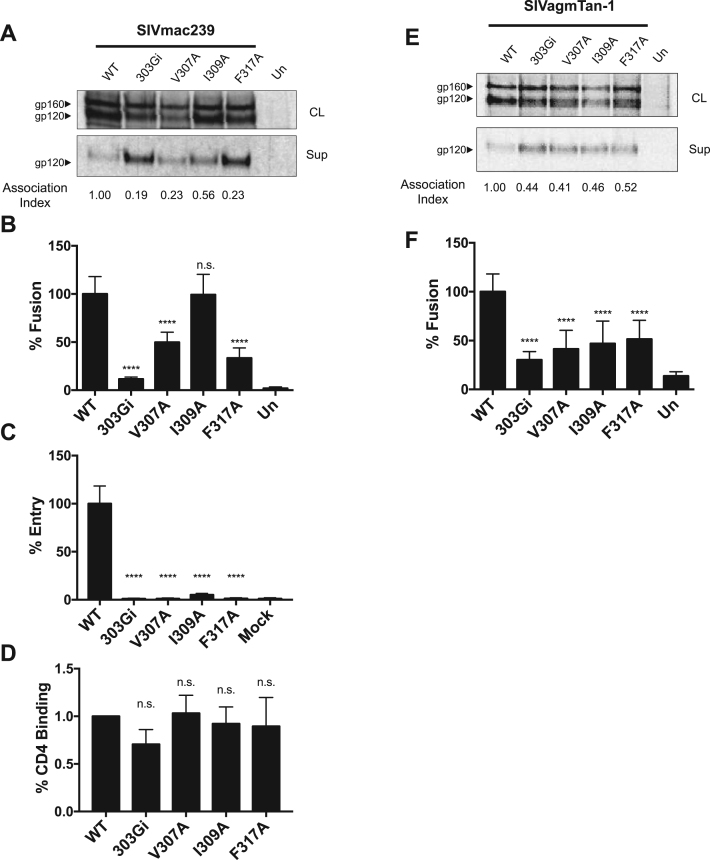
**Characterization of V3 hydrophobic-patch and glycine-insertion mutants of SIV strains.** (A). Immunoprecipitated cell lysates (CL) and supernatants (Sup) of glycine-insertion (303Gi) and hydrophobic-patch mutants of SIVmac239. The association index value was calculated as previously reported (Finzi et al., 2010). (B). Cell-cell fusion assay of SIVmac239 Env variants. (C). Viral entry of SIVmac239 Env variants. (E). Immunoprecipitated cell lysates (CL) and supernatants (Sup) of glycine-insertion and hydrophobic-patch mutants of SIVagmTan1. (F). Cell-cell fusion assay of SIVagmTan1 Env variants. Un, untransfected effector cells. Significance was determined by the student's T-test [* ** *p < 0.0001]; n.s., not significant.

Nonhuman primate viruses are the sources of HIV-1 and HIV-2 (26–30). Sequence comparisons suggest that the V3 hydrophobic patch is conserved in nonhuman lentiviruses (Fig. 1A). To determine whether the structural and functional roles of the V3 loop hydrophobic patch are conserved in non-human primate lentiviruses, we tested two typical SIV strains. We introduced a glycine insertion and alanine substitutions into the hydrophobic patch in the SIVmac239 envelope, and carried out the Env functional assays described above. Interestingly, in SIVmac239, the I309A change resulted in less shedding than the glycine insertion at position 303 or the V307A and the F317A changes (Fig. 6A), as reflected in a much higher association index. This trend correlated well with the cell-cell fusion and virus entry functions of these Envs, as fusion of I309A was not significantly different from that of the wild-type SIVmac239 Env (Fig. 6B); there was a detectable level of entry for the I309A viruses, although still significantly reduced, whereas the other Env mutants did not facilitate entry above the background level (Fig. 6C). The SIVmac239 Env appears to be more tolerant of an alanine residue at position 309 than at positions 307 and 317, and is better able to tolerate the alteration of residue 309 than the other primate lentiviruses studied in this work.

The binding of the SIVmac239 Env mutants to CD4-Ig was not significantly different from that of the WT Env (Fig. 6D); these results indicate that the introduced changes did not result in a major conformational change in the gp120 structure that affected binding to CD4.

We also altered the Env from another SIV strain, SIVagmTan1, inserting a glycine residue between residues 303 and 304, and substituting alanine residues for each of the residues associated with the hydrophobic patch. The phenotypes of the insertion mutant 303Gi and the three hydrophobic patch mutants (V307A, I309A and F317A) were consistent with those observed in the other viruses studied above. The introduced changes in the V3 loop resulted in decreased Env stability, indicated by increased gp120 shedding, and a corresponding decrease in the level of cell-cell fusion initiated by these mutant Envs (Fig. 6E, F). We note that the signal: background ratio for cell-cell fusion initiated by the SIVagmTan1 envelope was lower than that seen for the other Envs described above. This may be a result of the TZM-bl target cells expressing human, not African green monkey, CD4 and CCR5.

## 3 Discussion

The gp120 V3 loop of primate immunodeficiency virus Envs is typically 35 amino acid residues in length and can be divided into the base, stem and tip (4, 21). The amino acid sequences of the V3 base and tip are more conserved than that of the stem. Mutagenesis studies have implicated the V3 base and tip in contacting the CCR5 or CXCR4 coreceptors: the V3 base contributes to the interaction with the sulfated N-terminus of the coreceptor, whereas the V3 tip interacts with extracellular loop 2 (ECL2) of the coreceptor (Abayev et al., 2015, Arimont et al., 2017, Huang et al., 2007, Kufareva, 2016). Although the amino acid sequences of the V3 stem are less conserved, our previous study demonstrated that insertions of one or two glycine residues in the V3 stem severely compromised the function of Env from Clade B HIV-1 (Xiang et al., 2010). Part of this functional attenuation arises from decreased coreceptor binding, presumably as a result of disruption of the two-point contact of the V3 base and tip with the coreceptor. The V3 loop length also appears to have been constrained by the requirement for gp120 to associate non-covalently with the assembled Env trimer. Insertion of glycine residues in the V3 stem of Envs from Clade B HIV-1 resulted in shedding of gp120 from the Env trimer, suggesting that the V3 loop might contribute to the non-covalent association of gp120 with the pre-triggered Env trimer (Xiang et al., 2010). Further investigation revealed the importance of the hydrophobic patch in the V3 tip in maintaining the stability of the assembled Env trimer (Xiang et al., 2010). The association of gp120 with the Env trimer correlated with the cell-cell fusion activity of panels of Env mutants derived from a given primate immunodeficiency virus.

**Fig. 7:**
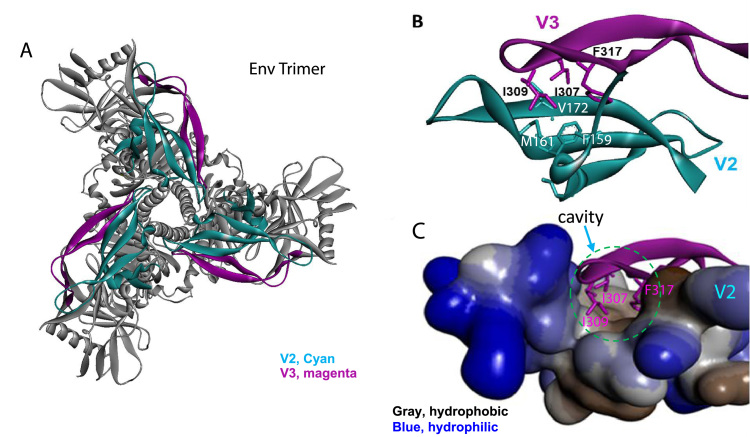
**Interaction of the gp120 V3 loop hydrophobic patch and V2 loop in the sgp140 SOSIP.664 Env trimer.** (A). HIV-1 sgp140 SOSIP.664 Env trimer structure in the unbound state (PDB: 4ZMJ). V3, magenta; V2, cyan. (B). Ribbon structure of V2 and V3 loops showing a key-in-hole interaction, with specific focus on the V3 hydrophobic patch residues at positions I307, I309 and F317 and with the hydrophobic cavity residues (i.e. F159, M161 and V172) of V2 loop. (C). V2 surface model showing the hydrophobic cavity (circled in cyan) interacting with the V3 loop hydrophobic patch. Gray, hydrophobic; blue, hydrophilic.

The results of our study indicate the general importance of the gp120 V3 loop hydrophobic patch in the subunit association of primate immunodeficiency virus Env trimers. Our observation that the stem insertion mutants and hydrophobic patch mutants exhibit similar phenotypes with respect to gp120 shedding implies that precise spatial interactions of the hydrophobic patch are required for Env trimer stability. On the available high-resolution structures of HIV-1 Env trimers (Julien et al., 2013a, Lee et al., 2016, Lyumkis et al., 2013, Pancera et al., 2014), the gp120 V1, V2 and V3 elements are located at the trimer apex (Fig. 7A). In these structures, the V3 hydrophobic patch forms a hydrophobic core by a key-in-hole interaction with the V2 region (F159, M161, and V172) of the same gp120 subunit (Fig. 7B and C). Alteration of the V3 and V2 hydrophobic interactions might destabilize structures near the Env trimer apex that contribute to maintenance of the pre-triggered conformation of the assembled trimer. The structural models suggest that the V2 turn (residues 164–168) immediately adjacent to the V3-V2 hydrophobic core is involved in interprotomer interactions in the Env trimer. An indirect disruption of these V2-mediated interprotomer interactions by alteration of the V3 hydrophobic patch might explain an “opening” of the trimer apex; such HIV-1 mutants would be expected to maintain trimer integrity and replication competence but exhibit increased neutralization sensitivity (Herschhorn et al., 2016). The observed shedding of gp120 resulting from the introduced V3 changes is more difficult to explain, as the gp120 subunits exhibit numerous interactions with gp41 in current Env trimer models (Julien et al., 2013a, Lee et al., 2016, Lyumkis et al., 2013, Pancera et al., 2014). That the gp120 shedding phenotypes of the V3 mutants result from indirect V2-mediated effects seems unlikely when the direct alteration of V2 residues 164–168 did not yield gp120 shedding phenotypes; in fact, most of these V2 mutants exhibited phenotypes similar to those of the wild-type virus (Herschhorn et al., 2016, Madani et al., 2017). Conformational differences between the high-resolution Env trimer structures and that of the pre-triggered Env on virions have been suggested (Alsahafi et al., 2015, Castillo-Menendez et al., 2018, Pancera et al., 2017). Therefore, in the pre-triggered virion Env trimer, the residues in the V3 hydrophobic patch may contribute to Env trimer stability through interactions not evident in current high-resolution structures. Additional structural information on the conformations sampled by the pre-triggered HIV-1 Env trimer may shed light on the mechanism whereby the V3 loop contributes to trimer stability.

**Fig. 8:**
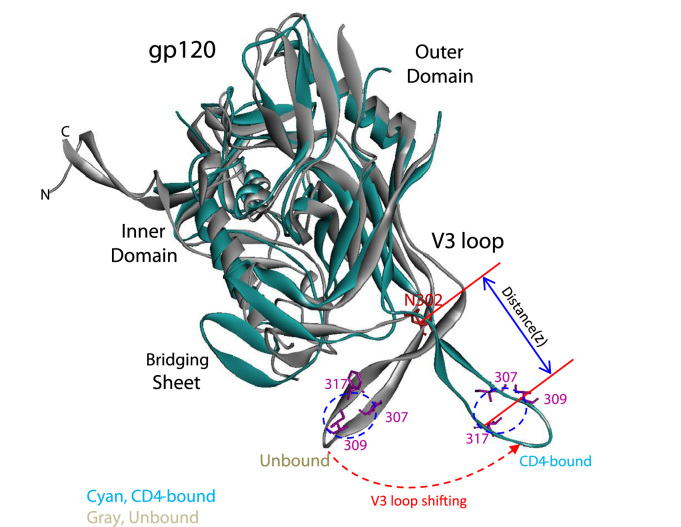
**Conformational changes in the gp120 V3 loop.** Model of superimposed gp120 structures without CD4 (gray) and CD4-bound (cyan) states, showing the shift in the position of the V3 loop. The V3 hydrophobic patch residues 307, 309 and 317 are shown in magenta. The V3 base residue N302 that interacts with the sulfated CCR5 N-terminus is also indicated in red. Part of the V2 loop in the unbound state is omitted for better visualization of the bridging sheet and V3 loop. The X-ray structures used for the superimposed structures are PDB: 4ZMJ (Kwon et al., 2015) for the unbound sgp140 SOSIP.664 state and PDB: 2QAD (Huang et al., 2007) for the CD4-bound state.

CD4 binding repositions the V3 loop from a relatively buried location to an extended structure that can interact with the CCR5 or CXCR4 chemokine receptors (Fig. 8). Coreceptor-binding sites in the V3 tip and base are made available for interaction with the chemokine receptor ECL2 and N-terminus, respectively. In this CD4-bound conformation, the distance (Z) between the V3 base and tip may be constrained by the distance between the N-terminal and ECL2 contacts on the chemokine receptor. In a previous report (Xiang et al., 2010), we found that the decreased syncytium-forming ability of some glycine-insertion V3 mutants could be partially compensated by a CCR5 mutant with an N-terminus extended by two glycine residues. The hydrophobic patch contributes to the interaction of the V3 tip with the chemokine receptor ECL2. Changing the hydrophobic patch residues in HIV-1 gp120 to alanine allowed partial CCR5 binding, but substitution of polar (serine) or charged (glutamic acid) residues abolished detectable CCR5 interaction. These results imply that hydrophobic contacts may be required for the efficient interaction of the V3 tip and ECL2 of the chemokine receptor. Differences in the amount of shed gp120 and in the utilization of monkey versus human receptors complicate quantitative comparisons of the relative contribution of the V3 hydrophobic patch to HIV-1/SIV and HIV-1 coreceptor interaction. However, from previous data derived with HIV-1 (Xiang et al., 2010), we expect that glycine insertions and reduction of hydrophobicity in the hydrophobic patch of the HIV-2/SIV gp120 V3 region will significantly decrease CCR5 binding.

Future studies should provide additional insights into the dual roles of the primate immunodeficiency virus V3 hydrophobic patch in maintenance of Env trimer stability and in coreceptor binding.

## 4 Methods and materials

### 4.1 Cell lines

293 T cells were used for Env expression experiments, as effector cells for the cell-cell fusion assay and to produce the single-round virus for entry assays. 293 T cells were grown in Dulbecco's modified Eagle Medium (DMEM) containing 10% fetal bovine serum, 8 mM L-glutamine and 100 μg/mL penicillin/streptomycin solution (complete DMEM). TZM-bl cells were grown in complete DMEM as described above, and were used as the target cells for the cell-cell fusion and virus entry assays. Cf2Th-CD4/CCR5 and Cf2Th-CCR5 were grown in complete DMEM; the Cf2Th-CD4/CCR5 cell medium was supplemented with 500 μg/mL G418 and 150 μg/mL Hygromycin B, and the Cf2Th-CCR5 cell medium was supplemented with 500 μg/mL G418.

### 4.2 Plasmids

Plasmids for envelope expression were generated by subcloning. The ZM249MB_10 (referred to as ZM249), HIV-2 ST and SIVagmTan1 envelope expression plasmids were generated by PCR amplification of the *env* genes from existing envelope expression plasmids or proviral constructs and ligation into pcDNA3.1+ for expression in mammalian cells. The envelope gene of ZM249 was amplified from the plasmid pZM249 (Parrish et al., 2013, Salazar-Gonzalez et al., 2009) by PCR using primers that added a 5’ HindIII site and 3’ EcoRI site. The HIV-2 ST envelope gene was amplified from the pHIV2/ST molecular clone (Kong et al., 1988, Kumar et al., 1990), adding a 5’ AflII site and a 3’ BamHI site, and subcloned into pcDNA3.1+. The SIVagmTan1 envelope gene was amplified from the pSIVagmTan-1 molecular clone (Soares et al., 1997), adding a KpnI site to the 5’ end of the gene and an EcoRI site to the 3’ end of the gene, followed by subcloning into pcDNA3.1 + . 1084i (Zhang et al., 2006) was subcloned into the pSVIIIenv plasmid expressing the HIV-1 HXBc2 Rev and Env proteins, using the KpnI site, which is conserved, and the BamHI site. A BamHI site corresponding to the existing BamHI site in HXBc2 was introduced into the 1084i sequence by site-directed mutagenesis. Following subcloning, all Env expressor constructs were sequenced to verify the integrity of the *env* gene. Mutations to alter the V3 loop hydrophobic patch were also made directly to ZM249 for pseudotyped virus production (Marcon et al., 1997, Xiang et al., 2002). All ligations were carried out using T4 DNA Ligase from New England Biolabs.

### 4.3 Site-directed mutagenesis

The V3 loop hydrophobic patch substitution and insertion mutants in HIV-1 subtype C strains (ZM249 and 1084i), HIV-2 ST, SIVagmTan1 and SIVmac239 were made by site-directed mutagenesis, using PfuUltra (Agilent) and the QuikChange XL protocol. The primers for mutagenesis were synthesized by Integrated DNA Technologies (IDT).

### 4.4 Cell-cell fusion assay

On Day 0, 3.5 × 10^5^ 293 T cells were plated in a 6-well plate. The following day, the cells were transfected with 2 μg of WT or mutant Env expression constructs and 1 μg Tat-expressing plasmid using polyethylynamine (PEI). Twenty-four hours following transfection, the medium was changed on the transfected cells, and TZM-bl cells were distributed in a black 96-well plate at a density of 1.0 × 10^4^ per well. On Day 3, the transfected 293 T cells were detached from the plate using gentle pipetting and counted in a hemocytometer. Approximately 1.0 × 10^4^ of the transfected effector cells were distributed into the wells containing the previously plated TZM-bl cells and the cells were co-incubated for 16 h. Following co-incubation, the cells were washed once with PBS, lysed using 1× Passive Lysis Buffer (Promega) and frozen at −80 °C. After thawing, he plates were read for luciferase activity using the beetle luciferin substrate (Promega) and a Veritas Luminometer. Values for the cell-cell fusion activities of WT Envs were set to 100% and mutant values are expressed as a percentage of the WT value.

### 4.5 Assay measuring Env processing and subunit association

293 T cells were set at a density of 3.5 × 10^5^ in a 6-well plate. The following day, the cells were transfected with WT or mutant Env expression construct. One day post transfection, the cells were metabolically labeled overnight using the EasyTag EXPRESS^35^S Protein Labeling Mix [^35^S] (PerkinElmer). Following labeling, the supernatants were harvested and clarified of cell debris by gentle centrifugation. Separately, the cells were lysed using a mild NP-40-based lysis buffer. The cell lysates were precleared using appropriate uninfected serum for 8 h as previously described (Bohl et al., 2013). Following the preclearing, the cell lysates and supernatant fractions were separately subjected to immunoprecipitation with serum from the corresponding virus-infected host, using Protein-A Sepharose beads (GE Health Sciences). The samples were run on SDS-PAGE and the gels were dried and exposed to a phosphor screen. Phosphor screens were developed using a Personal Molecular Imager (Biorad) and analyzed using QuantityOne software (Biorad). Values for the supernatant and cell lysate band were determined and the association index was calculated as previously described (Finzi et al., 2010).

### 4.6 Generation of pseudotyped viruses

293 T cells were plated at a density of 3.5 × 10^5^ cells per well in a 6-well plate. Twenty-four hours later, the cells were transfected with a WT or mutant Env expression plasmid and an *env*-deficient viral backbone (pSG3deltaEnv from the NIH AIDS Reagent Program (Wei et al., 2002, Wei et al., 2003) in the case of ZM249 and SIVmac239 constructs, or pHIV-1Luc and CMVPack for 1084i constructs) using PEI. One day following transfection, additional medium was added to the wells. Three days post transfection, the supernatants were harvested, briefly spun down to remove cell debris and stored at −80 °C. The pseudovirus-containing supernatants were then quantified using the reverse transcriptase assay.

### 4.7 Reverse transcriptase assay

To quantify the amount of pseudovirus in a sample, the reverse transcriptase assay was performed. In duplicate, 500 μL of pseudovirus-containing supernatant was spun at 14,000xg for 2 h at 4 °C. Following the spin, the supernatant was removed and the viral pellet was resuspended in a Triton X-100-based suspension buffer and vortexed, followed by a rapid freeze-thaw cycle a total of three times to lyse the virus. Fifty microliters of reaction mix (Oligo-dT Poly-A and ^3^H-dTTP (PerkinElmer)) was added, and the samples were incubated at 37 °C for 1 h in a heating block. Following incubation, the samples were pipetted onto cut squares of DEAE Filtermat paper (PerkinElmer), followed by three ten-minute washes in 2× SSC buffer, and one ten-second wash in 100% ethanol. The filters were dried at room temp and then analyzed using a scintillation counter to quantify the incorporation of ^3^H-dTTP into cDNA. The average CPM values from each duplicate were then used to normalize the amount of virus-containing supernatant that was used in subsequent single-round viral entry assays.

### 4.8 Single-round viral infection assay

Cf2Th-CD4/CCR5 or TZM-bl cells, which express both human CD4 and CCR5, were plated at a density of 8.0 × 10^3^ per well (Cf2Th-CD4/CCR5 cells were used in the case of 1084i Env-expressing pseudoviruses, and TZM-bl cells were used in the case of ZM249, ADA, JR-FL and SIVmac239 Env-expressing viruses). The following day, a volume of virus, normalized based on the level of RT activity in the supernatant, was added to each well in a final volume of 100 μL, and diluted in complete DMEM. One day after infection, the viral supernatants were removed, the cells were gently washed one time with PBS and fresh complete media was added to each well. On day 3 post infection, the supernatants were removed, the cells were washed once with PBS, and the cells were lysed in 1× Passive Lysis Buffer and frozen at −80 °C. The plates were then thawed and luciferase activity was measured using the Veritas Luminometer and beetle luciferin substrate (Promega).

### 4.9 CD4 binding assay

^35^S-labeled supernatants from cells expressing WT or mutant Envs were assessed for CD4 binding by performing an immunoprecipitation assay using human CD4-Ig. Input quantities were determined by performing an immunoprecipitation using the same volume of supernatant and serum from an appropriate virus-infected host. To measure the CD4 binding of HIV-2 ST variants, three times the volume of supernatant was used for comparison with the WT Env, because of a reduced level of binding to CD4-Ig compared to HIV-1 species. Following immunoprecipitation with CD4-Ig, the bands were quantified using Quantity One software (Biorad) and the volumes were compared to the volumes of the input gel. The ratio of WT CD4-Ig: serum immunoprecipitation was adjusted to 1, and the ratios for the mutants were compared to the WT values.

### 4.10 Statistical analysis

For all statistical analyses of cell-cell fusion and single-round viral infection assays, experimental groups were compared using the Student's T-test. Each experiment was performed three separate times, with quadruplicate values. Each mutant value was converted to a percentage of the WT value, which was set at 100%. Significant differences were determined using a *p*-value of ≤0.05. All statistical analysis was performed using GraphPad Prism 6 software.

## References

[bib1] Abayev M., Moseri A., Tchaicheeyan O., Kessler N., Arshava B., Naider F., Scherf T., Anglister J. (2015). An extended CCR5 ECL2 peptide forms a helix that binds HIV-1 gp120 through non-specific hydrophobic interactions. FEBS J..

[bib2] Alsahafi N., Debbeche O., Sodroski J., Finzi A. (2015). Effects of the I559P gp41 change on the conformation and function of the human immunodeficiency virus (HIV-1) membrane envelope glycoprotein trimer. PLoS One.

[bib3] Arimont M., Sun S.L., Leurs R., Smit M., de Esch I.J.P., de Graaf C. (2017). Structural analysis of chemokine receptor-ligand interactions. J. Med. Chem..

[bib4] Bohl C., Bowder D., Thompson J., Abrahamyan L., Gonzalez-Ramirez S., Mao Y., Sodroski J., Wood C., Xiang S.H. (2013). A twin-cysteine motif in the V2 region of gp120 is associated with SIV envelope trimer stabilization. PLoS One.

[bib5] Cardozo T., Kimura T., Philpott S., Weiser B., Burger H., Zolla-Pazner S. (2007). Structural basis for coreceptor selectivity by the HIV type 1 V3 loop. AIDS Res. Hum. Retrovir..

[bib6] Cashin K., Gray L.R., Jakobsen M.R., Sterjovski J., Churchill M.J., Gorry P.R. (2013). CoRSeqV3-C: a novel HIV-1 subtype C specific V3 sequence based coreceptor usage prediction algorithm. Retrovirology.

[bib7] Castillo-Menendez L., Witt K., Espy N., Princiotto A., Madani N., Pacheco B., Finzi A., Sodroski J. (2018). Comparison of uncleaved and mature Human Immunodeficiency Virus (HIV-1) membrane envelope glycoprotein trimers. J. Virol..

[bib8] Conley A.J., Conard P., Bondy S., Dolan C.A., Hannah J., Leanza W.J., Marburg S., Rivetna M., Rusiecki V.K., Sugg E.E. (1994). Immunogenicity of synthetic HIV-1gp120 V3-loop peptide-conjugate immunogens. Vaccine.

[bib9] de Taeye S.W., de la Pena A.T., Vecchione A., Scutigliani E., Sliepen K., Burger J.A., van der Woude P., Schorcht A., Schermer E.E., van Gils M.J., LaBranche C.C., Montefiori D.C., Wilson I.A., Moore J.P., Ward A.B., Sanders R.W. (2018). Stabilization of the gp120 V3 loop through hydrophobic interactions reduces the immunodominant V3-directed non-neutralizing response to HIV-1 envelope trimers. J. Biol. Chem..

[bib10] de Taeye S.W., Ozorowski G., Torrents de la Pena A., Guttman M., Julien J.P., van den Kerkhof T.L., Burger J.A., Pritchard L.K., Pugach P., Yasmeen A., Crampton J., Hu J., Bontjer I., Torres J.L., Arendt H., DeStefano J., Koff W.C., Schuitemaker H., Eggink D., Berkhout B., Dean H., LaBranche C., Crotty S., Crispin M., Montefiori D.C., Klasse P.J., Lee K.K., Moore J.P., Wilson I.A., Ward A.B., Sanders R.W. (2015). Immunogenicity of stabilized HIV-1 envelope trimers with reduced exposure of non-neutralizing epitopes. Cell.

[bib11] Finzi A., Xiang S.H., Pacheco B., Wang L., Haight J., Kassa A., Danek B., Pancera M., Kwong P.D., Sodroski J. (2010). Topological layers in the HIV-1 gp120 inner domain regulate gp41 interaction and CD4-triggered conformational transitions. Mol. Cell.

[bib12] Foda M., Harada S., Maeda Y. (2001). Role of V3 independent domains on a dualtropic human immunodeficiency virus type 1 (HIV-1) envelope gp120 in CCR5 coreceptor utilization and viral infectivity. Microbiol. Immunol..

[bib13] Hartley O., Klasse P.J., Sattentau Q.J., Moore J.P. (2005). V3: hiv's switch-hitter. AIDS Res. Hum. Retrovir..

[bib14] Herschhorn A., Ma X., Gu C., Ventura J.D., Castillo-Menendez L., Melillo B., Terry D.S., Smith A.B., Blanchard S.C., Munro J.B., Mothes W., Finzi A., Sodroski J. (2016). Release of gp120 restraints leads to an entry-competent intermediate state of the HIV-1 envelope glycoproteins. mBio.

[bib15] Hirsch V.M., Dapolito G., Goeken R., Campbell B.J. (1995). Phylogeny and natural history of the primate lentiviruses, SIV and HIV. Curr. Opin. Genet. Dev..

[bib16] Hongjaisee S., Braibant M., Barin F., Ngo-Giang-Huong N., Sirirungsi W., Samleerat T. (2017). Effect of amino acid substitutions within the V3 region of HIV-1 CRF01_AE on interaction with CCR5-coreceptor. AIDS Res. Human. Retrovir..

[bib17] Huang C.C., Lam S.N., Acharya P., Tang M., Xiang S.H., Hussan S.S., Stanfield R.L., Robinson J., Sodroski J., Wilson I.A., Wyatt R., Bewley C.A., Kwong P.D. (2007). Structures of the CCR5 N terminus and of a tyrosine-sulfated antibody with HIV-1 gp120 and CD4. Science.

[bib18] Huang C.C., Tang M., Zhang M.Y., Majeed S., Montabana E., Stanfield R.L., Dimitrov D.S., Korber B., Sodroski J., Wilson I.A., Wyatt R., Kwong P.D. (2005). Structure of a V3-containing HIV-1 gp120 core. Science.

[bib19] Jacob R.A., Moyo T., Schomaker M., Abrahams F., Grau Pujol B., Dorfman J.R. (2015). Anti-V3/glycan and Anti-MPER neutralizing antibodies, but not Anti-V2/glycan site antibodies, are strongly associated with greater Anti-HIV-1 neutralization breadth and potency. J. Virol..

[bib20] Jiang X., Burke V., Totrov M., Williams C., Cardozo T., Gorny M.K., Zolla-Pazner S., Kong X.P. (2010). Conserved structural elements in the V3 crown of HIV-1 gp120. Nat. Struct. Mol. Biol..

[bib21] Julien J.P., Cupo A., Sok D., Stanfield R.L., Lyumkis D., Deller M.C., Klasse P.J., Burton D.R., Sanders R.W., Moore J.P., Ward A.B., Wilson I.A. (2013). Crystal structure of a soluble cleaved HIV-1 envelope trimer. Science.

[bib22] Julien J.P., Sok D., Khayat R., Lee J.H., Doores K.J., Walker L.M., Ramos A., Diwanji D.C., Pejchal R., Cupo A., Katpally U., Depetris R.S., Stanfield R.L., McBride R., Marozsan A.J., Paulson J.C., Sanders R.W., Moore J.P., Burton D.R., Poignard P., Ward A.B., Wilson I.A. (2013). Broadly neutralizing antibody PGT121 allosterically modulates CD4 binding via recognition of the HIV-1 gp120 V3 base and multiple surrounding glycans. PLoS Pathog..

[bib23] Kong L.I., Lee S.W., Kappes J.C., Parkin J.S., Decker D., Hoxie J.A., Hahn B.H., Shaw G.M. (1988). West African HIV-2-related human retrovirus with attenuated cytopathicity. Science.

[bib24] Korber B., Foley B.T., Kuiken C., Pillai S.K., Sodroski J.G. (1998). Numbering positions in HIV relative to HXB2CG. Hum. Retrovir. AIDS.

[bib25] Kufareva I. (2016). Chemokines and their receptors: insights from molecular modeling and crystallography. Curr. Opin. Pharmacol..

[bib26] Kumar P., Hui H.X., Kappes J.C., Haggarty B.S., Hoxie J.A., Arya S.K., Shaw G.M., Hahn B.H. (1990). Molecular characterization of an attenuated human immunodeficiency virus type 2 isolate. J. Virol..

[bib27] Kumar R., Raghava G.P. (2013). Hybrid approach for predicting coreceptor used by HIV-1 from its V3 loop amino acid sequence. PLoS One.

[bib28] Kwon Y.D., Pancera M., Acharya P., Georgiev I.S., Crooks E.T., Gorman J., Joyce M.G., Guttman M., Ma X., Narpala S., Soto C., Terry D.S., Yang Y., Zhou T., Ahlsen G., Bailer R.T., Chambers M., Chuang G.Y., Doria-Rose N.A., Druz A., Hallen M.A., Harned A., Kirys T., Louder M.K., O'Dell S., Ofek G., Osawa K., Prabhakaran M., Sastry M., Stewart-Jones G.B., Stuckey J., Thomas P.V., Tittley T., Williams C., Zhang B., Zhao H., Zhou Z., Donald B.R., Lee L.K., Zolla-Pazner S., Baxa U., Schon A., Freire E., Shapiro L., Lee K.K., Arthos J., Munro J.B., Blanchard S.C., Mothes W., Binley J.M., McDermott A.B., Mascola J.R., Kwong P.D. (2015). Crystal structure, conformational fixation and entry-related interactions of mature ligand-free HIV-1 Env. Nat. Struct. Mol. Biol..

[bib29] Lee J.H., Ozorowski G., Ward A.B. (2016). Cryo-EM structure of a native, fully glycosylated, cleaved HIV-1 envelope trimer. Science.

[bib30] Li L., Wang X.H., Williams C., Volsky B., Steczko O., Seaman M.S., Luthra K., Nyambi P., Nadas A., Giudicelli V., Lefranc M.P., Zolla-Pazner S., Gorny M.K. (2015). A broad range of mutations in HIV-1 neutralizing human monoclonal antibodies specific for V2, V3, and the CD4 binding site. Mol. Immunol..

[bib31] Lyumkis D., Julien J.P., de Val N., Cupo A., Potter C.S., Klasse P.J., Burton D.R., Sanders R.W., Moore J.P., Carragher B., Wilson I.A., Ward A.B. (2013). Cryo-EM structure of a fully glycosylated soluble cleaved HIV-1 envelope trimer. Science.

[bib32] Madani N., Princiotto A.M., Zhao C., Jahanbakhshsefidi F., Mertens M., Herschhorn A., Melillo B., Smith A.B., Sodroski J. (2017). Activation and inactivation of primary human immunodeficiency virus envelope glycoprotein trimers by CD4-mimetic compounds. J. Virol..

[bib33] Marcon L., Choe H., Martin K.A., Farzan M., Ponath P.D., Wu L., Newman W., Gerard N., Gerard C., Sodroski J. (1997). Utilization of C-C chemokine receptor 5 by the envelope glycoproteins of a pathogenic simian immunodeficiency virus, SIVmac239. J. Virol..

[bib34] Moore J.P., Nara P.L. (1991). The role of the V3 loop of gp120 in HIV infection. Aids.

[bib35] Pancera M., Lai Y.T., Bylund T., Druz A., Narpala S., O'Dell S., Schon A., Bailer R.T., Chuang G.Y., Geng H., Louder M.K., Rawi R., Soumana D.I., Finzi A., Herschhorn A., Madani N., Sodroski J., Freire E., Langley D.R., Mascola J.R., McDermott A.B., Kwong P.D. (2017). Crystal structures of trimeric HIV envelope with entry inhibitors BMS-378806 and BMS-626529. Nat. Chem. Biol..

[bib36] Pancera M., Zhou T., Druz A., Georgiev I.S., Soto C., Gorman J., Huang J., Acharya P., Chuang G.Y., Ofek G., Stewart-Jones G.B., Stuckey J., Bailer R.T., Joyce M.G., Louder M.K., Tumba N., Yang Y., Zhang B., Cohen M.S., Haynes B.F., Mascola J.R., Morris L., Munro J.B., Blanchard S.C., Mothes W., Connors M., Kwong P.D. (2014). Structure and immune recognition of trimeric pre-fusion HIV-1 Env. Nature.

[bib37] Parrish N.F., Gao F., Li H., Giorgi E.E., Barbian H.J., Parrish E.H., Zajic L., Iyer S.S., Decker J.M., Kumar A., Hora B., Berg A., Cai F., Hopper J., Denny T.N., Ding H., Ochsenbauer C., Kappes J.C., Galimidi R.P., West A.P., Bjorkman P.J., Wilen C.B., Doms R.W., O'Brien M., Bhardwaj N., Borrow P., Haynes B.F., Muldoon M., Theiler J.P., Korber B., Shaw G.M., Hahn B.H. (2013). Phenotypic properties of transmitted founder HIV-1. Proc. Natl. Acad. Sci. USA.

[bib38] Salazar-Gonzalez J.F., Salazar M.G., Keele B.F., Learn G.H., Giorgi E.E., Li H., Decker J.M., Wang S., Baalwa J., Kraus M.H., Parrish N.F., Shaw K.S., Guffey M.B., Bar K.J., Davis K.L., Ochsenbauer-Jambor C., Kappes J.C., Saag M.S., Cohen M.S., Mulenga J., Derdeyn C.A., Allen S., Hunter E., Markowitz M., Hraber P., Perelson A.S., Bhattacharya T., Haynes B.F., Korber B.T., Hahn B.H., Shaw G.M. (2009). Genetic identity, biological phenotype, and evolutionary pathways of transmitted/founder viruses in acute and early HIV-1 infection. J. Exp. Med..

[bib39] Scheib H., Sperisen P., Hartley O. (2006). HIV-1 coreceptor selectivity: structural analogy between HIV-1 V3 regions and chemokine beta-hairpins is not the explanation. Structure.

[bib40] Sharp P.M., Hahn B.H. (2011). Origins of HIV and the AIDS Pandemic. Cold Spring Harb. Perspect. Med..

[bib41] Sirois S., Touaibia M., Chou K.C., Roy R. (2007). Glycosylation of HIV-1 gp120 V3 loop: towards the rational design of a synthetic carbohydrate vaccine. Curr. Med. Chem..

[bib42] Soares M.A., Robertson D.L., Hui H., Allan J.S., Shaw G.M., Hahn B.H. (1997). A full-length and replication-competent proviral clone of SIVAGM from tantalus monkeys. Virology.

[bib43] Stanfield R.L., Wilson I.A. (2005). Structural studies of human HIV-1 V3 antibodies. Hum. Antibodies.

[bib44] Svicher V., Alteri C., Artese A., Zhang J.M., Costa G., Mercurio F., D'Arrigo R., Alcaro S., Palu G., Clementi M., Zazzi M., Andreoni M., Antinori A., Lazzarin A., Ceccherini-Silberstein F., Perno C.F., group Os (2011). Identification and structural characterization of novel genetic elements in the HIV-1 V3 loop regulating coreceptor usage. Antivir. Ther..

[bib45] Totrov M., Jiang X., Kong X.P., Cohen S., Krachmarov C., Salomon A., Williams C., Seaman M.S., Abagyan R., Cardozo T., Gorny M.K., Wang S., Lu S., Pinter A., Zolla-Pazner S. (2010). Structure-guided design and immunological characterization of immunogens presenting the HIV-1 gp120 V3 loop on a CTB scaffold. Virology.

[bib46] Watkins J.D., Siddappa N.B., Lakhashe S.K., Humbert M., Sholukh A., Hemashettar G., Wong Y.L., Yoon J.K., Wang W., Novembre F.J., Villinger F., Ibegbu C., Patel K., Corti D., Agatic G., Vanzetta F., Bianchi S., Heeney J.L., Sallusto F., Lanzavecchia A., Ruprecht R.M. (2011). An anti-HIV-1 V3 loop antibody fully protects cross-clade and elicits T-cell immunity in macaques mucosally challenged with an R5 clade C SHIV. PLoS One.

[bib47] Wei X., Decker J.M., Liu H., Zhang Z., Arani R.B., Kilby J.M., Saag M.S., Wu X., Shaw G.M., Kappes J.C. (2002). Emergence of resistant human immunodeficiency virus type 1 in patients receiving fusion inhibitor (T-20) monotherapy. Antimicrob. Agents Chemother..

[bib48] Wei X., Decker J.M., Wang S., Hui H., Kappes J.C., Wu X., Salazar-Gonzalez J.F., Salazar M.G., Kilby J.M., Saag M.S., Komarova N.L., Nowak M.A., Hahn B.H., Kwong P.D., Shaw G.M. (2003). Antibody neutralization and escape by HIV-1. Nature.

[bib49] Xiang S.H., Finzi A., Pacheco B., Alexander K., Yuan W., Rizzuto C., Huang C.C., Kwong P.D., Sodroski J. (2010). A V3 loop-dependent gp120 element disrupted by CD4 binding stabilizes the human immunodeficiency virus envelope glycoprotein trimer. J. Virol..

[bib50] Xiang S.H., Kwong P.D., Gupta R., Rizzuto C.D., Casper D.J., Wyatt R., Wang L., Hendrickson W.A., Doyle M.L., Sodroski J. (2002). Mutagenic stabilization and/or disruption of a CD4-bound state reveals distinct conformations of the human immunodeficiency virus type 1gp120 envelope glycoprotein. J. Virol..

[bib51] Zhang H., Hoffmann F., He J., He X., Kankasa C., West J.T., Mitchell C.D., Ruprecht R.M., Orti G., Wood C. (2006). Characterization of HIV-1 subtype C envelope glycoproteins from perinatally infected children with different courses of disease. Retrovirology.

[bib52] Zolla-Pazner S. (2005). Improving on nature: focusing the immune response on the V3 loop. Hum. Antibodies.

